# CK2 Molecular Targeting—Tumor Cell-Specific Delivery of RNAi in Various Models of Cancer

**DOI:** 10.3390/ph10010025

**Published:** 2017-02-21

**Authors:** Janeen H. Trembley, Betsy T. Kren, Md. Joynal Abedin, Rachel I. Vogel, Claire M. Cannon, Gretchen M. Unger, Khalil Ahmed

**Affiliations:** 1 Research Service, Minneapolis VA Health Care System, Minneapolis, MN 55417, USA; krenx@umn.edu (B.T.K.); mdjoynal@yahoo.com (M.J.A.); ahmedk@umn.edu (K.A.); 2 Department of Laboratory Medicine and Pathology, University of Minnesota, Minneapolis, MN 55455, USA; 3 Masonic Cancer Center, University of Minnesota, Minneapolis, MN 55455, USA; isak0023@umn.edu; 4 Department of Obstetrics, Gynecology and Women’s Health, University of Minnesota, Minneapolis, MN 55455, USA; 5 School of Veterinary Medicine, University of Minnesota, Minneapolis, MN 55455, USA; clairemcannon@gmail.com; 6 GeneSegues Therapeutics, Minnetonka, MN 55343, USA; gmu@genesegues.com; 7 Department of Urology, University of Minnesota, Minneapolis, MN 55455, USA

**Keywords:** CK2, nanocapsules, nanoparticles, anti-CK2, RNAi, siRNA, tenfibgen, TBG, TBG-RNAi-CK2, therapy, cancer, targeting, cancer-specific, tumor-specific, prostate cancer, breast cancer, HNSCC

## Abstract

Protein kinase CK2 demonstrates increased protein expression relative to non-transformed cells in the majority of cancers that have been examined. The elevated levels of CK2 are involved in promoting not only continued proliferation of cancer cells but also their resistance to cell death; thus, CK2 has emerged as a plausible target for cancer therapy. Our focus has been to target CK2 catalytic subunits at the molecular level using RNA interference (RNAi) strategies to achieve their downregulation. The delivery of oligonucleotide therapeutic agents warrants that they are protected and are delivered specifically to cancer cells. The latter is particularly important since CK2 is a ubiquitous signal that is essential for survival. To achieve these goals, we have developed a nanocapsule that has the properties of delivering an anti-CK2 RNAi therapeutic cargo, in a protected manner, specifically to cancer cells. Tenfibgen (TBG) is used as the ligand to target tenascin-C receptors, which are elevated in cancer cells. This strategy is effective for inhibiting growth and inducing death in several types of xenograft tumors, and the nanocapsule elicits no safety concerns in animals. Further investigation of this therapeutic approach for its translation is warranted.

## 1. Introduction

Protein kinase CK2 (acronym for the former casein kinase II or 2) is a ubiquitous protein serine/threonine kinase consisting of two catalytic subunits (42 kDa α and 38 kDa α’) linked through two regulatory subunits (28 kDa β). The kinase is present in the nuclear and cytoplasmic fractions of the cell, including numerous cellular organelles. The distribution of the kinase in normal cells is diffuse in both the nuclear and cytoplasmic compartments; however, in cancer cells the overall expression and nuclear localization of CK2 is significantly enhanced [1,2,3,4].

Over time, several investigations recognized the association of protein kinase CK2 with cell growth and proliferation in normal and cancer cells (reviewed in, e.g., [1,4,5,6,7]). However, its link to cancer biology was firmly established only when it was discovered that CK2 not only promotes cell growth and proliferation but also is an effective suppressor of cell death, originally revealed by this laboratory [8,9,10,11]. Likewise, it was recognized that the treatment of cells with antisense to CK2 resulted in potent cell death, thus prompting the notion, for the first time, that CK2 downregulation might serve as cancer therapy strategy [12]. The potential of CK2 as a druggable target for cancer therapy has been documented with several recent studies on its targeting primarily using small molecular inhibitors or by peptide-mediated inhibition of its phosphorylation site(s), (see, e.g., [13,14,15]).

## 2. Discussion

### 2.1. Features of CK2 Pertinent to Cancer

CK2 is present in all cells and its activity appears to be stable at a certain level depending on the organ. CK2 (activity/level) is elevated in cells during proliferation, and in normal cell proliferation the CK2 activity returns to the basal level on cessation of proliferation. The basal CK2 activity is involved in a wide range of cellular activities as has been reviewed in several publications (e.g., [2,3]). The possible involvement of CK2 in cancer is supported by a wide range of observations. First, CK2 has been found to be elevated in the vast majority of cancers that have been examined [5,7,16]. Its elevation, however, is not simply an indicator of cell proliferation but rather indicates a state of dysplasia [17] and contributes to transformation [18,19,20]. The intracellular distribution of CK2 proteins in cancer cells is also distinct from that in normal cells such that the level of CK2 in the nuclear compartment of cancer cells is much higher than that in normal cells [7,17,21].

Second, CK2 is a potent regulator of cell death such that its elevation serves as a potent suppressor of apoptosis while its downregulation results in the induction of cell death—this characteristic of CK2 may be regarded as the key functional link of this enzyme to the cancer cell phenotype [5,8,9,10]. A number of hallmarks of cancer have been described [22]. However, two consistent characteristics of cancer cells are a dysregulation of cell growth and proliferation as well as a resistance to cell death. Thus, it is reasonable to surmise that up-regulation of CK2 in cancer cells not only provides the environment for their continued proliferation but also contributes to their suppression of cell death [8,9,10,23]. Considering the dysregulation of CK2 in all cancers that have been examined, we previously presented discussions of the studies suggesting involvement of CK2 in several of the hallmarks of cancer (besides the two mentioned above) [24,25]. It is also important to note that the level of CK2 dysregulation in a given cancer has been linked to prognosis (e.g., [26,27,28,29,30]). Here we present further discussion on the roles for CK2 in proliferation and malignancy, and the results of molecular targeting of CK2 in cancer, based on our recent research publications.

### 2.2. CK2 Elevation in Benign Prostate Proliferation versus in Prostate Cancer

The aforementioned observations describe that CK2 elevation has been uniformly observed in various cancers. In general, CK2 level/activity is elevated during normal cell proliferation as well [1,4,31]; however, there are cases of benign pathologic cell proliferation where CK2 is present at an elevated level as observed, e.g., in benign prostatic hyperplasia (BPH) [27,30,32]. This raises a question as to the nature of functionality of CK2 in prostatic cancer (PCa) versus BPH. To address this issue, we examined the combined expression of CK2α and NFκB p65 in PCa and BPH, considering that both are elevated in each of these diseases and that NFκB p65 is a known substrate of CK2α [33,34]. We thus wanted to determine if there was a differential expression of CK2α and NFκB p65 that may provide a means of distinction between the two diseases. An analysis of both of these signals in human PCa and BPH specimens revealed a number of observations. First, the amount of nuclear staining for CK2α and NFκB p65 was much higher in PCa than BPH. Second, expression levels of nuclear NFκB p65 and nuclear CK2α were correlated in both disease states. Correlation between CK2α and NFκB p65 expression observed at the protein level was generally not evident at the mRNA level in PCa based on comparison of mRNA expression for CSNK2A1 and RELA (information for mRNA expression in BPH was not readily available [35,36,37]). Third, increased nuclear CK2α protein expression in cancer was at least partially responsible for increased nuclear localization of NFκB p65 in PCa. Fourth, we found that PCa specimens demonstrated higher proliferation (Ki-67 immunostaining) than BPH specimens. Interestingly, nuclear NFκB p65 levels correlated with cytoplasmic NFκB p65 as well as Ki-67 signals in BPH, but not in PCa. It is noteworthy that these various correlations were apparent at the protein level and require the ability to determine nuclear and cytoplasmic localization of the signals [33]. Together, these observations suggest that with respect to their combined activity CK2 and NFκB have different modes of functionality in the biology of benign prostate growth and malignancy.

### 2.3. CK2 as a Target of Cancer Therapy

As mentioned, CK2 expression and activity are altered in most cancers that have been examined, and its molecular downregulation results in potent induction of cell death in cancer cells in culture or in vivo [7,12,38,39]. These observations from our laboratory prompted us to propose the notion that CK2 may serve as a target for cancer therapy, provided means for its effective targeting are developed (discussed subsequently). Since that time, CK2 has gained general acceptance as a target for cancer therapy, and currently diverse methods to achieve this targeting are being investigated [40,41]. Among the various approaches to targeting CK2 are the use of small molecule inhibitors, use of a peptide to block CK2 phosphorylation sites, and molecular downregulation employing RNAi to catalytic subunits of CK2 [13,42,43,44,45,46,47,48]. CK2 as a therapy target is particularly appealing because of its potent and rapid effect on activation of the apoptotic machinery [49], while also likely affecting the activity of a large number of downstream intracellular substrates and pathways as a consequence of its downregulation [6,50].

The nature of CK2 as ubiquitous and essential for cell survival has raised concerns regarding its targeting for cancer therapy, as its inhibition or loss might result in considerable toxicity to the host [12]. However, it has been observed that normal cells are significantly more resistant to CK2 inhibition or molecular downregulation [38,44]. These observations would suggest a pharmacological window for using CK2 as a target for cancer therapy. Regardless, we have been focused on the therapeutic targeting of CK2 in a cancer cell-specific manner, as discussed subsequently.

### 2.4. Desirability of Targeting CK2 Specifically in Cancer Cells and Utility of the TBG Nanocapsule to Accomplish This Goal

The relative resistance of normal cells to downregulation of CK2 has prompted considerable interest in the possible utility of small molecule inhibitors (such as CX-4945) as therapeutic agents. However, since CK2 is a ubiquitous survival signal, it would seem appropriate to employ strategies that are specific for downregulating CK2 in cancer cells, avoiding normal cells in vivo. Secondly, cancer cells may eventually develop drug resistance by various mechanisms, as has been a common observation in cancer chemotherapy (see, e.g., [22,51]). To avoid such a situation for CK2 targeted therapy, we have focused on blocking the production of CK2 by employing anti-CK2 RNAi strategies designed to block the generation of the two catalytic subunits. Such an approach warrants that systemic RNAi delivery is achieved in a protected manner and further that this delivery mechanism is cancer cell-specific. To that end, we have developed a nanocapsule that satisfies these requirements.

**Figure 1:**
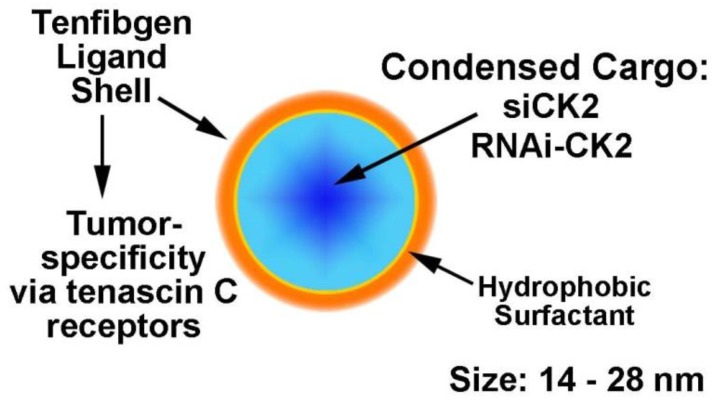
Nanocapsule concept and design and functional aspects are depicted.

Our nanocapsule is based on a protein shell containing tenfibgen (TBG) peptide which is the C-terminal fibrinogen globe domain of the 225 kDa fibronectin-like extracellular matrix protein tenascin-C (TN-C) (Figure 1, with permission of Springer [52]). The preparation of the TBG nanocapsules incorporating various types of anti-CK2 oligonucleotide molecules as well as dysprosium (Dy) has been described previously [47,48,53,54]. These nanocapsules are generally between 14 to 28 nm in size, and display uniform morphology (appearing as uniform single capsules) as determined by atomic force microscopy and transmission electron microscopy. They carry neutral or slightly negatively charge. The targeting of the TBG nanocapsules is based on the knowledge that TN-C receptors are elevated in cancer cells and thus the TBG nanocapsules hone to cancer cells but not to normal/benign cells [55,56,57,58,59,60,61]; this malignant cell specificity has been demonstrated in cultured cells as well as in xenograft models of PCa, breast cancer, and head and neck squamous cell carcinoma (HNSCC) [24,47,48,54,62,63,64]. The uptake of the nanocapsules into the cancer cells is via the lipid raft pathway [47]. Of note, the TBG nanocapsules are also capable of delivering their cargo effectively to tumor cells metastasized to lymph node and spleen [47,64]. A microscopy approach using TBG-encapsulated Dy (TBG-Dy), which takes advantage of the innate fluorescence of this lanthanide element, established that the nanocapsule was detected in xenograft tumor localized to tibia but not in normal counterpart tibia [64]. Further, analysis of several normal tissues in tumor-bearing animals treated with various types of TBG nanocapsules demonstrated the accumulation of the nanocapsule in primary and metastatic tumors, but not in tissue such as liver, brain, and kidney where no tumor was present, consistent with an absence of nonspecific uptake or trapping of the nanocapsule [40,47,63,64].

### 2.5. Response of Xenograft Tumors to TBG-RNAi-CK2

We investigated CK2 molecular downregulation for several types of cancer xenograft models by utilizing TBG nanocapsule based delivery of anti-CK2 oligomers (such as double-stranded siRNA or single-stranded RNAi) that target both of the catalytic subunits of CK2. Comparison of oligomer types was carried out because it is possible that single-stranded or double-stranded RNAi oligomers might show differential efficacy in vivo as they might distinctively employ lalternate RNAi processing mechanisms. For controls in these studies, TBG nanocapsules carrying non-targeted siRNA (TBG-siCON1) or carrying oligomers targeting mouse factor VII (TBG-RNAi-F7) were employed [46]. The siCON1 control oligomer is a non-targeting double-stranded siRNA, which was rigorously established [65]. The mouse factor VII sequence was chosen as a negative control for single-stranded oligomer as this sequence was previously shown to engage the RNAi machinery in mouse liver and because factor VII is not expressed in prostate tumor [66]. Studies on acute dose-response in prostate PC3-LN4 xenograft tumors suggested that TBG-RNAi-CK2 was most effective at reducing tumor growth in the dose range of 0.01 to 0.1 mg/kg given three times over a period of 10 days. Under similar experimental conditions, the TBG-siCK2 nanocapsules demonstrated the most effective dose level to be 0.01 mg/kg. In the castration-resistant prostate cancer (CRPC) 22Rv1 model, the most effective dose of TBG-RNAi-CK2 was 0.1 mg/kg. TBG-siCK2 treatment using 0.01 mg/kg also reduced tumor growth in the MDA-MB-231 model of triple negative breast cancer [48]. In all of these studies, the nanocapsule was delivered by intravenous tail vein injection. These data are summarized in Table 1.

**Figure 2:**
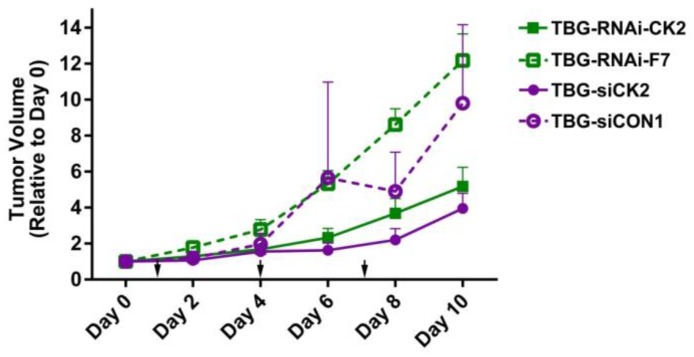
Comparison of xenograft tumor volumes over time in anti-CK2 and control TBG nanocapsule treated mice. The changes in PC3-LN4 tumor volumes relative to day 0 are shown following three nanocapsule drug treatments. Nanocapsule doses were 0.01 mg/kg for anti-CK2 and F7 nanocapsules and 1 mg/kg for TBG-siCON1 nanocapsules. Means + standard error of the mean are presented. Group sizes TBG-RNAi-CK2 *n* = 9; TBG-RNAi-F7 *n* = 8; TBG-siCK2 *n* = 9. TBG-siCON1 *n* = 8. Arrows indicate days that nanocapsule treatment injections occurred. Statistical significance for day 10 is given in Table 1.

In examining the tumor volume changes over time for both TBG-RNAi-CK2 and TBG-siCK2 dose response studies in the PC3-LN3 model, we noted that the time period from days 5 through 7 showed a dramatic separation in tumor growth rates between the anti-CK2 treated and the control treated mice (Figure 2). We set up another therapy study in which mice were treated with TBG-RNAi-CK2 or TBG-RNAi-F7 nanocapsules at 0.01 mg/kg on days 1 and 4, and tumors were collected on days 5, 6 and 7. These tumors were then used to examine the molecular and cellular responses within the treated tumors over the time course.

**Figure 3:**
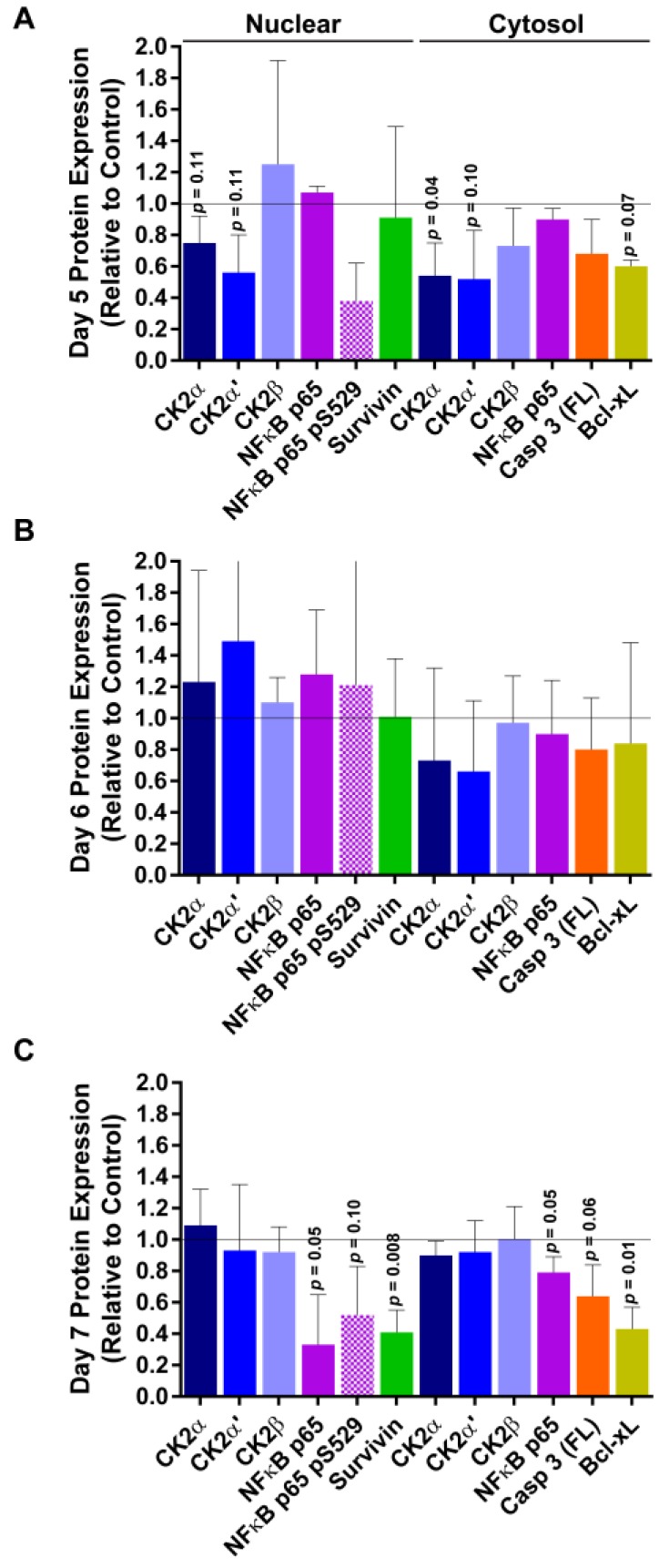
Protein expression response over time to TBG-RNAi-CK2 treatment in PC3-LN4 xenograft tumors. Immunoblot analysis of nuclear and cytosol fractionated PC3-LN4 tumor lysates following intravenous treatments of 0.01 mg/kg TBG-RNAi-CK2 or TBG-RNAi-F7 was performed. Protein signals were quantitated and means and standard deviations are graphed for (**A**) day 5, (**B**) day 6, and (**C**) day 7 following initiation of nanocapsule treatments. Group sizes TBG-RNAi-CK2 *n* = 3; TBG-RNAi-F7 *n* = 6 (days 5 and 6) or 7 (day 7). The grey line at expression level of “1” marks the expression for control treated tumors.

After performing immunoblot analyses on nuclear and cytosolic fractions from the time course tumors, we noted a biphasic response (Figure 3). On day 5, 24 h following the TBG-RNAi-CK2 treatment on day 4, CK2α and CK2α’ protein levels were notably reduced in both the nuclear and cytosolic compartments. At the same time, there was loss of NFκB p65 phosphorylation on S529, a CK2 phosphorylation site, as well as decreased levels of full length caspase 3 and the survival protein Bcl-xL. These markers indicate that some cell death was occurring, as well as loss of CK2 signaling. On day 6, there were decreased CK2α and CK2α’ protein levels in cytosol, although not statistically significant. On day 7, 72 h after TBG-RNAi-CK2 treatment, a second wave of cell death markers was noted. These markers included loss of total NFκB p65 as well as NFκB p65 P-S529, decreased nuclear survivin and cytosolic Bcl-xL. A further marker was loss of pro-caspase 3, suggesting probable activation of caspase 3 through cleavage. In addition to these events, immunohistochemical Ki-67 analysis demonstrated a dramatic decreased in proliferative cells in day 7 TBG-RNAi-CK2-treated tumors relative to day 5 tumors [46].

In the TBG-RNAi-CK2 dose response studies using the prostate models PC3-LN4 and 22Rv1, several interesting observations were made [46]. For example, we observed that the higher dose level (0.1 mg/kg) required for best repression of 22Rv1 tumor growth compared with PC3-LN4 tumor response (0.01 mg/kg) related to the higher levels of caveolin 1 in PC3-LN4 tumors compared with that in 22Rv1 tumors, suggesting more effective uptake of the drug in the PC3-LN4 model. Other factors influencing the tumor response to TBG-RNAi-CK2 in these two prostate models related to the levels of argonaute 1 (Ago 1), Ago 2, and GW182, which were present in higher amounts in the PC3-LN4 xenograft tumors compared with 22Rv1. In these in vivo studies of the TBG-RNAi-CK2 therapy of xenograft tumors, there was no evidence of the uptake of the nanocapsules in non-cancer tissues such as liver and spleen. Likewise, there was no evidence of a change in the blood serum chemistry for urea nitrogen, creatinine, total serum protein, alanine aminotransferase, and aspartate aminotransferase. Finally, no tissue damage to liver, spleen, or kidney has been observed in multiple studies [46,63].

Direct quantitative analysis of the entry of TBG nanocapsules into tumor cells was undertaken by testing the uptake of TBG encapsulated dysprosium (TBG-Dy). The presence of dysprosium cargo (a fluorescent lanthanide element) in dissociated tumor cells was detected by fluorescence activated cell sorting (FACS) analysis. The results revealed that more than 46% of the cells demonstrated the presence of the nanocapsules in LNCaP orthotopic tumor cells examined at 20 h after just one nanocapsule injection via either intravenous or intraperitoneal routes [46]. Uptake of TBG-Dy nanocapsules was also analyzed by FACS in two models of triple negative breast cancer, demonstrating an average of 33.9% positive cells in SUM-149 tumors and 11% positive cells in one MDA-MB-231 tumor [48].

**Figure 4:**
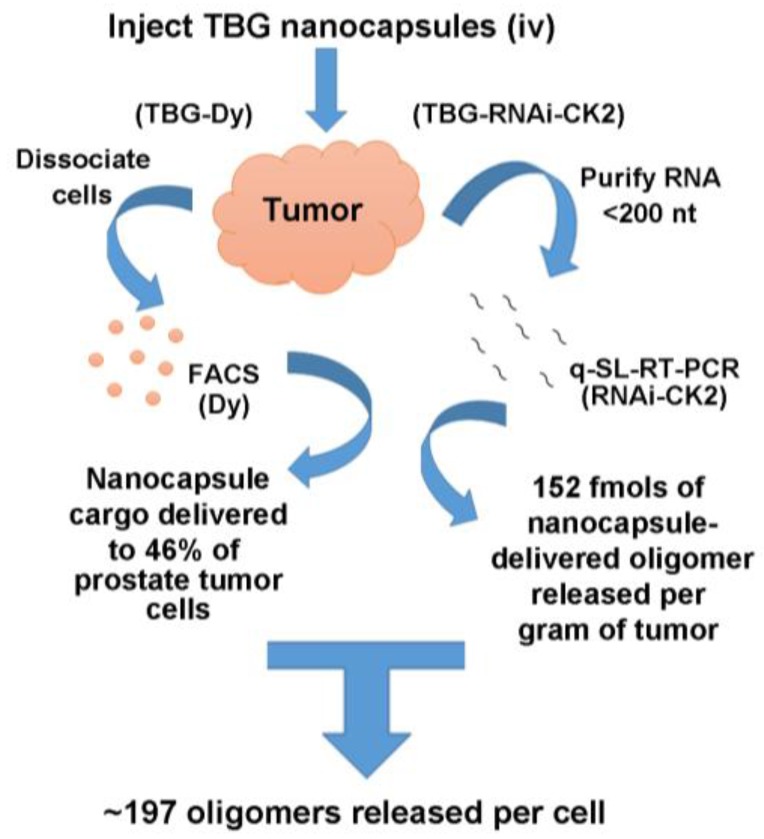
Quantitative analyses for TBG nanocapsule delivery to tumor and for release of RNAi-CK2 oligomer within tumors. Mice carrying LNCaP orthotopic xenograft tumors were injected via tail vein or intraperitoneal routes with TBG-Dy nanocapsules. The tumors were collected 20 h post-injection, the cells were dissociated, and the Dy signal was detected by FACS. For detection of oligomer within tumors, mice carrying PC3-LN4 xenograft flank tumors were treated once by tail vein injection at 1 mg/kg dose. Twenty-four hours after injection, RNAi-CK2 oligomer with a standard 3’-OH chemistry was detected by quantitative stem-loop RT-PCR on RNA purified from tissues. The number of oligomers released per cell was based on the mean percentage of Dy(+) prostate cells per tumor, the mean fmols of oligomer released per gram of tumor, and the mean theoretical number of cells per gram of tumor as 10^9^. q-SL-RT-PCR, quantitative stem-loop reverse transcriptase polymerase chain reaction. FACS, fluorescence activated cell sorting.

A complimentary analysis was undertaken to quantify the amount of bioavailable oligomer that is released into tumor cells following nanocapsule entry and proteolytic breakdown. In this study, mice carrying PC3-LN4 xenograft flank tumors were injected with TBG-RNAi-CK2 nanocapsules or with a combination of naked RNAi-CK2 oligomer plus TBG-erythritol. The naked oligomer/sugar nanocapsule combination was used to formally test that the RNAi-CK2 oligomers are entering tumor cells due to TBG encapsulation and not due to non-specific association with TBG. Released RNAi-CK2 oligomer was measured in RNA purified from tumor homogenates using quantitative stem-loop reverse transcriptase PCR. The results demonstrated that six of six tumors from mice receiving TBG-RNAi-CK2 contained an average of 152 fmols of released oligomer per gram of tumor. In contrast, none of the six tumors from mice receiving naked RNAi-CK2/TBG-erythritol contained detectable oligomer, indicating the utility and necessity of the TBG encapsulation. Taking the TBG-Dy uptake data together with the oligomer release data, and using the generally accepted value of 10^9^ cells per gram of tumor, we calculate that approximately 197 RNAi-CK2 oligomers were contained per tumor cell. The nanocapsule uptake and oligomer release data from prostate cancer xenograft tumors is summarized in Figure 4.

The above-described studies clearly established the potential of the TBG-based nanocapsule as a means of delivering the anti-CK2 RNAi therapy in a protected and cancer-cell-specific manner. We have also examined the effects of TBG nanoencapsulated anti-CK2 therapy in HNSCC. Similar to the data discussed for prostate and breast cancer, short-term studies in HNSCC demonstrated the efficacy of TBG nanoencapsulated RNAi-CK2 in multiple models of HNSCC [62]. A significant reduction in tumor growth was demonstrated concordant with modulation of CK2 targets including NFκB regulated molecules, TP53 protein, and apoptosis pathway proteins [62]. In follow-up studies, evidence was produced to show that TBG nanocapsule-delivered RNAi-CK2 co-opted the Ago 2/RISC pathway to target the CK2α/α’ mRNAs. The TBG-RNAi-CK2 nanocapsules reduced the growth of three different xenograft models of HNSCC affecting not only the primary tumor but also the metastases [47]. In these studies, a 6-month host survival was achieved at relatively low doses of the therapeutic agent without any apparent adverse effect in normal tissues, further supporting the translational potential of the TBG-RNAi-CK2 therapeutic for diverse cancers.

### 2.6. Phase I Trial of TBG-RNAi-CK2 in Large Animal Patients

Recently, we demonstrated that feline oral squamous cell carcinoma (FOSCC) and feline breast cancer cell lines have high expression of CK2 and that downregulation of CK2 in these cells resulted in induction of apoptosis analogous to observations on various cancer cell lines of human origin [7,67]. For the TBG-RNAi-CK2 nanocapsule to enter human clinical translation, an important step is to test its effects in large animals. FOSCC is commonly observed in domestic cats and has many features in common with human HNSCC; accordingly, FOSCC has been proposed as a large animal model for human HNSCC [68]. We decided to undertake a preclinical phase I trial of TBG-RNAi-CK2 use in FOSCC patients. The primary goal of this trial was to evaluate safety of the nanocapsule use, and a secondary goal was to assess for evidence of anti-CK2 targeting and tumor response. RNAi-CK2 oligomers were designed to specifically target feline CK2α and α’ and were formulated into TBG nanocapsules. Our study involved nine FOSCC patients with advanced disease and these patients were given two intravenous treatments per week of the therapeutic agent over a total period of three weeks. Two dose levels of the therapeutic were employed for two groups divided between the nine enrolled cats. Tumor and normal oral mucosal tissue biopsies were collected zero to seven days before treatment was initiated and again three to four days after the last treatment. Tumors were measured at treatment initiation and completion, and blood was collected for analyses at treatment start, once per week during treatment, and after treatment ceased. Tumor response was evaluated by RECIST criteria. Results from this initial large animal study are promising (our unpublished data), and together with the mouse studies provide a strong impetus for the pursuit of the TBG-RNAi-CK2 based nanomedicine approach for further studies to enter human clinical trials.

## 3. Conclusions

We have provided a brief overview of our work that contributed to the linking of CK2 to the cancer cell phenotype. Overexpression of CK2 in cancer cells at the protein level is a consistent observation, and much evidence now supports the notion that CK2 is a desirable target for cancer therapy. The elevation of CK2 in benign proliferation, as observed in BPH compared with PCa, suggests a complex role and possible involvement of other signals (such as NFκB p65) in the biology of CK2 in benign growth versus cancer cell growth. Since CK2 is essential for cell survival, one would surmise that loss of its physical presence and/or its lack of function would result in an effective mode of cancer therapy. Chemical or molecular downregulation of CK2 results in potent induction of cell death in cancer cells but normal cells tend to be somewhat resistant to downregulation of CK2, suggesting a possible therapeutic window that may be utilized for use of small molecule inhibitors of CK2 as chemotherapeutic agents. However, therapeutic targeting of CK2 through its molecular downregulation (using RNAi methods) warrants that the anti-CK2 therapeutic agent delivery is achieved in a protected and cancer cell-specific manner. Towards that end, we have described the application of a TBG nanocapsule that has the properties of delivering CK2 RNAi oligomers to primary and metastatic sites in several types of xenograft tumor models resulting in inhibition of tumor growth. The TBG-RNAi-CK2 drug has essentially no adverse effects in normal cells, and in a small study of naturally occurring feline oral cancer we found the drug to be well tolerated without any serious adverse events. Our studies on PCa therapy have highlighted the observation that downregulation of CK2 is effective in treatment of the therapy resistant form of prostate cancer, namely, CRPC, thus highlighting the utility of this therapeutic approach to hormonal cancers regardless of the activity of various receptors present therein.

**Figure 5:**
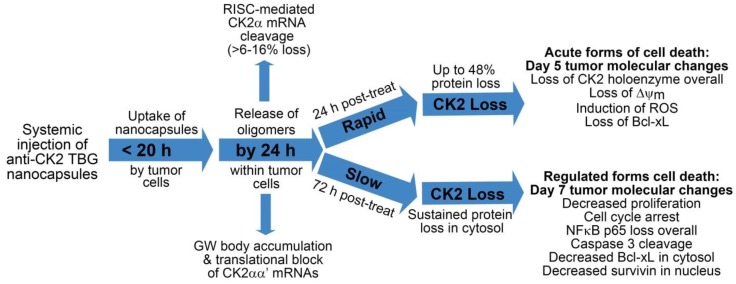
Flow chart illustrating proposed timeline of response and mechanisms of drug action and cell death within tumors. Notes: translational block of CK2αα’ mRNAs is inferred; loss of ∆ψ m (mitochondrial membrane potential) and induction of ROS in tumors on day 5 are based on data in cultured cells, not tumor data; decreased proliferation is based on published Ki-67 data in tumors; cell cycle arrest is based on cultured cell data.

Based on our observations in cultured cells, where precise and/or very short timed effects are more feasible, and in xenograft tumor models, we propose that loss of CK2 holoenzyme proteins and/or enzymatic activity induces cell death in at least two ways. First, acute loss of CK2 function (greater than 30% loss) causes rapid induction of cell death via decreased mitochondrial health, induction of reactive oxygen species (ROS), and loss of survival proteins such as Bcl-xL. Second, sustained but less severe loss of CK2 function over time causes cell cycle arrest, decreased proliferative signaling such as NFκB p65 activation and localization, induction of cell death through loss of survival proteins such as survivin and Bcl-xL, and through caspase 3 activation. This general theory on the function of the TBG-CK2-RNAi nanocapsules as a cancer therapeutic is described in the following flow diagram (Figure 5), and references for this diagram are found here [46,47,48,49,62,63,64,69,70,71,72,73,74,75,76].

Thus, based on the various observations described in the foregoing we propose that the TBG nanocapsule delivery of RNAi-CK2 specifically to cancer cells merits further consideration as a therapeutic agent for various cancers. Future work will broaden our elucidation of the timing and steps in death pathway mechanisms induced by acute and chronic CK2 signaling loss.

## Figures and Tables

**Table 1:** Comparison of oligomer-based anti-CK2 TBG nanocapsule effects on tumor volumes in multiple cancer models.

Tumor Model	Treatment	Tumor Volume on Final Day ^a^	*p*-Value
PC3-LN4	TBG-RNAi-CK2—0.01 mg/kg	5.2 ± 3.2	0.005
	TBG-RNAi-F7—0.01 mg/kg	12.2 ± 4.2	
PC3-LN4	TBG-siCK2—0.01 mg/kg	4.0 ± 2.5	0.007
	TBG-siCON1—1.0 mg/kg	10.6 ± 5.5	
22Rv1	TBG-RNAi-CK2—0.1 mg/kg	2.5 ± 1.5	0.11
	TBG-RNAi-F7—1.0 mg/kg	4.0 ± 1.5	
MDA-MB-231	TBG-siCK2—0.01 mg/kg	1.4 ± 0.32	0.026
	TBG-siCON1—0.01 mg/kg	2.1 ± 0.55	

^a^ Tumor volume at sacrifice (days 10 or 11) relative to start of treatment (mean ± standard deviation).
